# Association of Diet Quality With Survival Among People With Metastatic Colorectal Cancer in the Cancer and Leukemia B and Southwest Oncology Group 80405 Trial

**DOI:** 10.1001/jamanetworkopen.2020.23500

**Published:** 2020-10-30

**Authors:** Erin L. Van Blarigan, Sui Zhang, Fang-Shu Ou, Alan Venlo, Kimmie Ng, Chloe Atreya, Katherine Van Loon, Donna Niedzwiecki, Edward Giovannucci, Eric G. Wolfe, Heinz-Josef Lenz, Federico Innocenti, Bert H. O’Neil, James E. Shaw, Blase N. Polite, Howard S. Hochster, James N. Atkins, Richard M. Goldberg, Robert J. Mayer, Charles D. Blanke, Eileen M. O’Reilly, Charles S. Fuchs, Jeffrey A. Meyerhardt

**Affiliations:** 1 Department of Epidemiology and Biostatistics, University of California, San Francisco; 2 Dana-Farber/Partners CancerCare, Boston, Massachusetts; 3 Alliance Statistics and Data Management Center, Mayo Clinic, Rochester, Minnesota; 4 Department of Medicine, University of California, San Francisco; 5 Alliance Statistics and Data Center, Duke University, Durham, North Carolina; 6 Department of Nutrition and Department of Epidemiology, Harvard T.H. Chan School of Public Health, Boston, Massachusetts; 7 USC Norris Comprehensive Cancer Center, University of Southern California, Los Angeles; 8 Division of Pharmacotherapy and Experimental Therapeutics, UNC Eshelman School of Pharmacy; Department of Medicine—Hematology, University of North Carolina at Chapel Hill; 9 Simon Cancer Center, Indiana University School of Medicine, Indianapolis; 10 Virginia Commonwealth University, Richmond; 11 Pritzker School of Medicine, The University of Chicago, Chicago, Illinois; 12 Department of Medical Oncology, Yale University School of Medicine, New Haven, Connecticut; 13 Southeast Clinical Oncology Research Consortium, Winston-Salem, North Carolina; 14 West Virginia University Cancer Institute, Morgantown; 15 SWOG Group Chair’s Office, Knight Cancer Institute, Oregon Health & Science University, Portland; 16 Memorial Sloan Kettering Cancer Center, New York, New York; 17 Yale Cancer Center, Yale School of Medicine, New Haven, Connecticut

## Abstract

**Importance:**

Diet has been associated with survival in patients with stage I to III colorectal cancer, but data on patients with metastatic colorectal cancer are limited.

**Objective:**

To examine the association between diet quality and overall survival among individuals with metastatic colorectal cancer.

**Design, Setting, and Participants:**

This was a prospective cohort study of patients with metastatic colorectal cancer who were enrolled in the Cancer and Leukemia Group B (Alliance) and Southwest Oncology Group 80405 trial between October 27, 2005, and February 29, 2012, and followed up through January 2018.

**Exposures:**

Participants completed a validated food frequency questionnaire within 4 weeks after initiation of first-line treatment for metastatic colorectal cancer. Diets were categorized according to the Alternative Healthy Eating Index (AHEI), Alternate Mediterranean Diet (AMED) score, Dietary Approaches to Stop Hypertension (DASH) score, and Western and prudent dietary patterns derived using principal component analysis. Participants were categorized into sex-specific quintiles.

**Main Outcomes and Measures:**

Multivariable hazard ratios (HRs) and 95% CIs for overall survival.

**Results:**

In this cohort study of 1284 individuals with metastatic colorectal cancer, the median age was 59 (interquartile range [IQR]: 51-68) years, median body mass index was 27.2 (IQR, 24.1-31.4), 521 (41%) were female, and 1102 (86%) were White. There were 1100 deaths during a median follow-up of 73 months (IQR, 64-87 months). We observed an inverse association between the AMED score and risk of death (HR quintile 5 vs quintile 1, 0.83; 95% CI, 0.67-1.04; *P* = .04 for trend), but the point estimates were not statistically significant. None of the other diet scores or patterns were associated with overall survival.

**Conclusions and Relevance:**

In this prospective analysis of patients with metastatic colorectal cancer, diet quality assessed at initiation of first-line treatment for metastatic disease was not associated with overall survival.

## Key Points

**Question:**

Is diet quality at initiation of first-line treatment for metastatic colorectal cancer associated with overall survival?

**Findings:**

In this cohort study including 1284 people with metastatic colorectal cancer enrolled between 2005 and 2012, diet quality (assessed by the Alternative Healthy Eating Index, Alternate Mediterranean Diet score, Dietary Approaches to Stop Hypertension diet score, a Western dietary pattern, and a prudent dietary pattern) was not associated with overall survival after a median follow-up of 73 months.

**Meaning:**

The results of this study suggest that overall diet quality assessed at initiation of first-line treatment for metastatic colorectal cancer was not associated with overall survival.

## Teaser

This cohort study of patients with metastatic colorectal cancer examines associations between diet at initiation of first-line treatment and overall survival.

## Introduction

Colorectal cancer is the second leading cause of cancer death in the US. Approximately 22% of patients have metastatic disease at diagnosis, and 5-year survival for these individuals is only 14%.^1^ Thus, there is a need to identify factors associated with survival of patients with metastatic colorectal cancer.

Among people with stage I to III colorectal cancer, a Western dietary pattern,^2^ high glycemic load,^3^ and sugar-sweetened beverages^4^ are associated with higher risk of recurrence and death, whereas long-chain n-3 fatty acids,^5,6,7^ dark fish,^5^ fiber,^8^ whole grains,^9^ nuts,^10^ and coffee^11^ are associated with lower risk. No studies have examined diet in relation to survival among individuals with metastatic colorectal cancer.

Therefore, we examined overall diet quality and patterns assessed at the start of initial treatment for metastatic colorectal cancer in relation to overall survival. Our primary hypothesis was that a Western dietary pattern would be associated with shorter overall survival in people with metastatic colorectal cancer.^2^

## Methods

### Study Design and Patients

This cohort study used data from individuals who were enrolled in Cancer and Leukemia Group B (now part of Alliance for Clinical Trials in Oncology)/Southwest Oncology Group 80405 [CALGB/SWOG 80405]),^12^ a National Cancer Institute–sponsored trial of first-line therapy for advanced or metastatic colorectal cancer, between October 27, 2005, and February 29, 2012. A total of 2334 patients were enrolled. Institutional review board approval was required from all participating sites and each participant signed an institutional review board–approved, protocol-specific informed consent document in accordance with federal and institutional guidelines. Diet in association with outcomes was embedded as part of the original protocol from 2005, and every subsequent version of the protocol included diet as a secondary objective that received institutional review board approval. Eligibility criteria, recruitment procedures, and outcomes of the study have been previously reported^12^; briefly, participants had to be 18 years or older, have untreated locally advanced or metastatic colorectal cancer, have an Eastern Cooperative Oncology Group performance status of 0 or 1, and have normal laboratory values (eg, hepatic, renal, or hematologic). Patients were excluded if they had undergone major surgery in the previous 4 weeks or minor surgery in the previous 2 weeks. Prior adjuvant chemotherapy (up to 6 months) must have been completed at least 12 months before recurrence. Patients in the data set were deidentified. There was no difference in survival between treatment arms. This study followed the Strengthening the Reporting of Observational Studies in Epidemiology (STROBE) reporting guideline.^13^

An optional survey including a validated food frequency questionnaire (FFQ)^14,15,16,17,18^ was completed by 1354 participants within 4 weeks after treatment initiation; this subset had similar characteristics to the total study population. To reduce measurement error in diet, we excluded patients missing more than 70 items on the FFQ, as well as women with an estimated caloric intake less than 500 or greater than 3500 kcal per day and men with estimated caloric intake less than 600 or greater than 4200 kcal per day, which resulted in 1284 individuals who were eligible for analysis.

### Dietary Assessment

The FFQ assessed average intake of specified portions of approximately 130 items during the past 3 months, using as many as 9 response options, ranging from 0 to 6 or more servings per day.^14,15,16,17,18^ We computed nutrient intake by multiplying the frequency of consumption of each food by the nutrient content of the specified portion using values from the US Department of Agriculture.^19^ We adjusted for energy using the residual method.^20^

### Diet Quality Scores

We assessed 3 diet quality scores and 2 dietary patterns, as previously described.^21^ The Alternative Health Eating Index (AHEI) is scored from 0 to 110 and is based on vegetables (excluding potatoes), fruits, whole grains, nuts and legumes, long-chain n-3 fatty acids, polyunsaturated fatty acids, sweetened beverages and juice, red and processed meat, trans fat, sodium, and alcoholic drinks. The Alternative Mediterranean Diet (AMED) is scored from 0 to 9 and is based on vegetables, fruits, nuts, whole grains, legumes, fish, ratio of monounsaturated to saturated fat, red and processed meat, and alcohol. The Dietary Approaches to Stop Hypertension (DASH) is scored from 0 to 45 and is based on fruits, vegetables, nuts and legumes, low-fat dairy, whole grains, sodium, sweetened beverages, red and processed meats, and sweets and desserts. Higher scores indicate healthier diets for these 3 scores.

Principal component analysis was used to identify common dietary patterns based on the FFQ data collected in our study population, as previously described.^2^ The Western dietary pattern was characterized by higher intake of dairy, refined grains, condiments, red meat, and sweets and desserts. The prudent pattern was characterized by high intake of vegetables, legumes, and fruit. Dietary pattern scores were generated for each participant based on intake of each item and the item’s factor loading and standardized to mean 0 and standard deviation 1. Higher scores indicate diets more consistent with that specific pattern.

### Covariates 

Comprehensive data on potential confounding factors were obtained through medical record review and the participant questionnaire. Clinical data (eg, performance status, tumor location, prior resection, prior treatment for nonmetastatic disease, and a history of diabetes) and sociodemographic factors (age, sex, and race/ethnicity) were obtained from the medical record. Methods to determine *KRAS* status of participants’ tumors have been previously described.^12,22^ Lifestyle factors, including body mass index (BMI; calculated as weight in kilograms divided by height in meters squared), weight change in the past 6 months, and physical activity,^23,24^ were obtained through the questionnaire.

### Main Outcome and Follow-up

Our primary outcome was death from any cause.^12^ Data collection occurred continuously from 2005 until the data set was locked in January 2018.

### Statistical Analysis

Participants in CALGB/SWOG 80405 were pooled and analyzed as a prospective cohort. We used multivariable Cox proportional hazards regression to estimate hazard ratios (HRs) and 95% CIs.^25,26,27^ Overall survival was defined as time from completion of the FFQ until death or the end of follow-up.

We categorized the diet quality scores and patterns into sex-specific quintiles. Our basic model included age (years), sex, and daily energy intake (kcal). Our second model was additionally adjusted for race/ethnicity, performance status, protocol chemotherapy, primary tumor unresected, diabetes, treatment arm, *KRAS*,^12,22^ tumor sidedness, weight change in the previous 6 months, BMI, and weekly total physical activity (metabolic equivalent task hours per week). A missing indicator was used to account for missing data in *KRAS* and tumor sidedness. One individual was missing data on BMI, 3 were missing data on physical activity, and 20 were missing data on weight change in the previous 6 months; these were assigned to the most common categories for multivariate adjustment. We evaluated the proportional hazards assumption using the Schoenfeld residual method.^28^ To test for evidence of a linear trend (*P* trend), we modeled the median values of the diet score and pattern quintiles as ordinal variables in the multivariate Cox proportional hazards regression models and used a Wald test.^29,30^ We also explored whether associations differed by age (<60 vs ≥60 years), sex, tumor sidedness (right or transverse vs left), *KRAS* (wild type vs variant), performance status (0 vs 1 or 2), BMI (<21, 21-24.9, 25-29.9, 30-34.9, ≥30), and diabetes status (yes vs no). For each, we added a cross-product term between the potential modifier and exposure of interest to our multivariate models and used Wald tests to evaluate whether there was evidence that associations varied by level of the modifier.

Reverse causation bias may occur if individuals change their diet before death because of their underlying illness. To assess potential bias resulting from reverse causation, we explored whether results changed when events that occurred 90 days or less after FFQ administration (n = 135; 12%) were excluded. This approach applies a minimum time between the exposure assessment and outcome ascertainment and thus reduces the potential for the outcome to influence the exposure.

Two-sided *P* values <.05 were considered statistically significant. Statistical analyses were conducted using SAS version 9.4 (SAS Institute Inc).

## Results

In this cohort study of 1284 individuals with metastatic colorectal cancer, the median age was 59 (interquartile range [IQR], 51-68) years, the median BMI was 27.2 (IQR, 24.1-31.4), 521 (41%) were female, and 1102 (86%) were White (Table 1). There were 1100 deaths (86%) during a median follow-up of 73 (IQR, 64-87) months; nearly all deaths (976 [89%]) in this patient population were attributable to colorectal cancer.

**Table 1.** Sociodemographic, Clinical, and Lifestyle Characteristics of 1284 Individuals With Metastatic Colorectal Cancer

Characteristic	No. (%)
Age, median (IQR), y	59 (51**-**68)
Female	521 (41)
White race	1102 (86)
ECOG performance status	
0	795 (62)
1	488 (38)
2	1 (0)
Tumor sidedness^a^	
Right or transverse	448 (35)
Left	741 (58)
Unknown	95 (7)
Chemotherapy regimen	
Leucovorin, fluorouracil, irinotecan (FOLFIRI)	295 (23)
Leucovorin, fluorouracil, oxaliplatin (mFOLFOX6)	989 (77)
*KRAS* status^b^	
Wild type	768 (60)
Variant	285 (22)
Unknown	231 (18)
Treatment arm	
Bevacizumab	495 (39)
Cetuximab	481 (38)
Both	308 (24)
Primary tumor unresected	268 (21)
Prior radiation therapy	108 (8)
Prior adjuvant chemotherapy	168 (13)
Diabetes	221 (17)
BMI, median (IQR)	27.2 (24.1**-**31.4)
Physical activity, median (IQR), MET-h/wk	3.4 (0.6**-**12.7)
Calories, median (IQR), kcal/d	1814 (1369**-**2345)

Abbreviations: BMI, body mass index (calculated as weight in kilograms divided by height in meters squared); ECOG, Eastern Cooperative Oncology Group; IQR, interquartile range; MET, metabolic equivalent task.

^a^ Right-sided: cecum to hepatic flexure; transverse: hepatic to splenic flexure; left-sided: splenic flexure to rectum.

^b^ Expanded *KRAS* covering codons 12, 13, 19, 22, 61, 68, 117, and 146.^12,22^

Overall, none of the diet scores or patterns examined were associated with survival in metastatic colorectal cancer (Table 2). We observed an inverse association between the AMED score and risk of death (HR quintile 5 [Q5] vs quintile 1 [Q1], 0.83; 95% CI, 0.67-1.04; *P* = .04 for trend), but point estimates were not statistically significant. Additionally, the Western diet pattern was associated with longer survival in individuals with *KRAS* variant tumors (HR Q5 vs Q1, 0.50; 95% CI, 0.32-0.77) but not those with wild-type tumors (HR Q5 vs Q1, 0.95; 95% CI, 0.68-1.33) (*P*  = .02 for interaction). None of the other diet scores or patterns were associated with survival, overall or in subgroups. Results did not change when patients who died within 90 days after administration of the FFQ were excluded.

**Table 2.** Association Between Diet Quality Assessed at the Time of Treatment Initiation for Metastatic Disease and Overall Survival Among 1284 People With Metastatic Colorectal Cancer^a^

	Sex-specific quintile, HR (95% CI)					*P* value for trend^b^
	1	2	3	4	5	
**AHEI score**						
Events, No.	218	225	216	226	215	
Model 1^c^	1 [Reference]	0.97 (0.80-1.17)	0.91 (0.76-1.11)	1.08 (0.90-1.30)	0.84 (0.70-1.02)	.17
Model 2^d^	1 [Reference]	0.91 (0.75-1.09)	0.98 (0.81-1.19)	1.16 (0.96-1.41)	0.88 (0.73-1.08)	.68
**AMED score**						
Events, No.	278	186	192	292	152	
Model 1^c^	1 [Reference]	0.88 (0.73-1.06)	0.94 (0.78-1.14)	0.81 (0.68-0.96)	0.82 (0.67-1.02)	.02
Model 2^d^	1 [Reference]	0.92 (0.76-1.11)	0.94 (0.77-1.14)	0.83 (0.70-0.99)	0.83 (0.67-1.04)	.04
**DASH score**						
Events, No.	220	206	252	211	211	
Model 1^c^	1 [Reference]	0.88 (0.73-1.06)	0.84 (0.70-1.01)	0.94 (0.77-1.13)	0.85 (0.71-1.04)	.26
Model 2^d^	1 [Reference]	0.87 (0.71-1.05)	0.89 (0.74-1.07)	0.96 (0.79-1.17)	0.91 (0.75-1.12)	.75
**Prudent dietary pattern**						
Events, No.	220	225	225	221	209	
Model 1^c^	1 [Reference]	0.91 (0.75-1.09)	0.87 (0.72-1.06)	0.89 (0.73-1.09)	0.77 (0.62-0.96)	.03
Model 2^d^	1 [Reference]	0.98 (0.81-1.18)	0.94 (0.77-1.14)	0.93 (0.76-1.14)	0.83 (0.66-1.04)	.08
**Western dietary pattern**						
Events	214	223	229	221	213	
Model 1^c^	1 [Reference]	1.07 (0.88-1.29)	1.01 (0.83-1.23)	1.01 (0.81-1.26)	0.90 (0.70-1.16)	.28
Model 2^d^	1 [Reference]	1.08 (0.89-1.31)	1.04 (0.85-1.28)	0.99 (0.79-1.23)	0.85 (0.65-1.10)	.12

Abbreviations: AHEI, Alternative Healthy Eating Index; AMED, Alternative Mediterranean Diet; DASH, Dietary Approaches to Stop Hypertension; HR, hazard ratio.

^a^ Higher scores indicate healthier diets for all except the Western dietary pattern.

^b^ *P* value for trend calculated by modeling the sex-specific quintile medians of each category as a continuous term.

^c^ Model 1: Cox proportional hazards regression adjusted for age, sex, and kcal/d.

^d^ Model 2: Cox proportional hazards regression adjusted for variables in Model 1 plus race/ethnicity; performance status; protocol chemotherapy; primary tumor unresected; diabetes; treatment arm; *KRAS* status; tumor sidedness; weight change in previous 6 months; body mass index, calculated as weight in kilograms divided by height in meters squared (<21, 21-24.9, 25-29.9, 30-34.9, ≥35); and physical activity, measured by metabolic equivalent task hours per week (<3, 3-8.9, 9-17.9, ≥18).

## Discussion

In this prospective cohort study of 1284 individuals with metastatic colorectal cancer, there was no statistically significant association between diet quality or pattern assessed at initiation of first-line treatment for metastatic disease and overall survival.

Patients and clinicians often seek advice on whether diet changes or other modifiable factors can impact outcomes. Although there are increasing data on modifiable factors, such as exercise and diet, in people with colorectal cancer, there remains a paucity of data to guide patients with advanced and metastatic disease. In a recent study with this same cohort of patients,^24^ physical activity was observed to be associated with longer survival. In contrast, the current study did not find an association between overall diet quality and survival. Thus, although data are limited to 1 cohort so far, efforts to help patients adopt and maintain a physical activity routine may be more important than suggesting changes to their overall dietary pattern at the time of initiation of treatment for metastatic colorectal cancer.

### Strengths and Limitations

This study has strengths, including its large sample size and number of events, comprehensive lifestyle and treatment data, and complete follow-up.

The study also has limitations. There was only a one-time, baseline measure of diet. Consequently, these results may have been attenuated by nondifferential measurement error in diet, and it remains possible that diet before or after initiation of first-line treatment for metastatic disease may affect survival in these patients. In addition, reverse causation is a concern; people who feel ill before death may change their diet. However, all participants in this study had adequate bone marrow, liver, and renal function at enrollment and all but 1 had an Eastern Cooperative Oncology Group performance status of 0 or 1 when they completed the FFQ. Furthermore, the results did not change when patients who died within 90 days after the FFQ were excluded. A final limitation is that the majority of participants in this study were White, and the results may not be generalizable to people of other races/ethnicities.

## Conclusions

Diet quality and patterns at initiation of first-line therapy for metastatic colorectal cancer were not associated with overall survival in this large prospective cohort study. Studies with repeated measures of diet, as well as studies examining diet in relation to quality of life, among patients with metastatic colorectal cancer are needed. Research examining diet in relation to colorectal cancer survival in more diverse populations is also needed.
